# GLP‐1 at the Metabolic–Cognitive Interface: Reward, Affect, and Memory

**DOI:** 10.1002/cph4.70129

**Published:** 2026-03-24

**Authors:** Serena X. Gao, Léa Décarie‐Spain, Cindy Gu, Giulia Mazzini, Scott E. Kanoski, Tito Borner

**Affiliations:** ^1^ Department of Biological Sciences University of Southern California Los Angeles California USA; ^2^ Neuroscience Graduate Program University of Southern California Los Angeles California USA; ^3^ Department of Nutrition University of Montreal Montreal Quebec Canada; ^4^ Institute of Veterinary Physiology, Vetsuisse Faculty University of Zurich Zurich Switzerland

## Abstract

Glucagon‐like peptide‐1 (GLP‐1) is a nutrient‐responsive hormone classically associated with glucose homeostasis and food intake control, yet its receptor is broadly expressed throughout the central nervous system (CNS) in circuits governing complex cognitive processes. Here, we synthesize emerging evidence from preclinical models and human studies demonstrating that GLP‐1 receptor (GLP‐1R) signaling modulates multiple cognitive domains, including reward and motivational processes relevant to obesity and substance use disorder, affective‐related behaviors, and learning and memory. We propose a unifying framework in which GLP‐1R signaling acts as a key interoceptive indicator of energy status, dynamically modulating cognitive and behavioral output in accordance with metabolic state. In animal models, GLP‐1R activation dampens effort‐based seeking for palatable food and addictive drugs alike, exerts bidirectional effects on affective behavior (e.g., anxiety‐like behavior), and promotes synaptic plasticity, learning, and neuroprotection. Clinical studies further indicate that GLP‐1R agonists alter neural responses to reward‐related cues, influence mood‐related outcomes, and are associated with reduced risk of cognitive decline, although results pertaining to benefits in neurodegenerative disease remain mixed. Collectively, these data position GLP‐1R signaling as a metabolic‐cognitive interface linking internal energy status to reward processing, affective regulation, and memory, and highlight the importance of disentangling direct central actions from indirect secondary metabolic effects when evaluating the therapeutic potential of GLP‐1‐based interventions for psychiatric and cognitive disorders.

## Graphical abstract

GLP‐1R signaling integrates metabolic state with neural circuits controlling reward, mood, and memory, acting as a metabolic‐cognitive interface. In reward pathways, it suppresses hedonic feeding and drug seeking; in affective circuits, sustained signaling promotes anxiolytic and antidepressant effects; and in the hippocampus, it enhances synaptic plasticity and supports learning and memory.

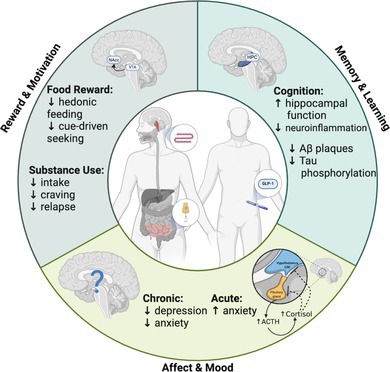

## 1 Introduction

Introduced first in 2005, glucagon‐like peptide‐1 (GLP‐1) analogs have since rapidly revolutionized the treatment of Type 2 diabetes mellitus (T2DM) and obesity by providing effective glycemic control in tandem with significant weight loss. Currently, GLP‐1 receptor (GLP‐1R) agonism—alone or with co‐agonism—represents the most effective pharmacological treatment approach for weight loss (see (Baggio and Drucker 2021; Müller et al. 2019) for review). Semaglutide, a long‐acting GLP‐1R agonist (GLP‐1RA), is the first FDA‐approved medication to show double‐digit weight loss in people with overweight/obesity without T2DM (Wilding et al. 2021), reflecting the efficacy of this class of drugs in addressing one of the most pressing global health challenges—the obesity epidemic.

While the beneficial effects of GLP‐1R agonists on glucose homeostasis are mediated in part by direct activation of GLP‐1R expressed on pancreatic β‐cells and on vagal afferent terminals (see (Drucker 2006; Hayes et al. 2014) for review), GLP‐1Rs are also widely expressed in the central nervous system (CNS), where central receptor engagement contributes substantially to the food intake‐ and body weight‐reducing effects of GLP‐1RAs such as Exendin‐4 (Ex‐4), liraglutide, and semaglutide (Adams et al. 2018; Alhadeff et al. 2017; Barrera et al. 2009; Fortin et al. 2020; Gabery et al. 2021; Hayes et al. 2008, 2011; Kanoski et al. 2011; Secher et al. 2014; Sisley et al. 2014). Consistent with this notion, a substantial body of literature from rodent models demonstrates that direct CNS administration of GLP‐1RAs robustly suppresses food intake and promotes weight loss, whereas pharmacological or genetic blockade/disruption of central GLP‐1R signaling yields opposing effects (see (Müller et al. 2019; Kanoski et al. 2016) for review).

GLP‐1R expression in the brain is not limited to regions predominantly involved in metabolic and satiation control (e.g., hypothalamic and hindbrain regions), but rather, is densely expressed throughout the brain in regions that are traditionally associated with higher‐order cognitive processes. For example, mesolimbic “reward” structures such as the ventral tegmental area (VTA) and nucleus accumbens (ACB; aka, NAc), together with cortical and hippocampal regions implicated in emotion, cognition, and memory all densely express GLP‐1Rs. Through these interconnected circuits, GLP‐1RAs are well positioned to influence reward processing, mood regulation, learning and memory, and other higher‐order cognitive processes. In the following sections, we review emerging evidence for GLP‐1R modulation of (1) reward and motivation, including for both food and addictive substances, (2) affective and anxiety disorders, and (3) learning and memory. We highlight mechanistic insights from preclinical rodent models, as well as from human clinical studies to provide a comprehensive framework for understanding the neurobiological mechanisms through which GLP‐1RAs may be beneficial for the treatments of substance use, affective, and memory disorders.

## 2 Reward and Motivation

### 2.1 Preclinical Evidence

Reward and motivation are fundamental aspects of survival, driving organisms to seek out stimuli that are essential or that otherwise provide pleasure, such as palatable food and addictive drugs, respectively. Central to these processes are dopaminergic circuits in the brain, particularly within the midbrain (VTA) and striatum (ACB). These regions are critical for processing reward signals, and dopamine (DA) plays a key role in positively reinforcing environmental stimuli and behavioral actions. Dysregulation of these reward pathways is a hallmark of major public health epidemics, including obesity and substance use disorders. In these conditions, palatable foods and drugs possess strong reinforcing properties that promote craving and drive repeated consumption. Here, we review emerging literature examining how GLP‐1R activation in animal models shapes reward‐driven behaviors, with a focus on its modulation of mesolimbic circuitry.

#### 2.1.1 Food Intake

An accumulating body of research demonstrates that GLP‐1R signaling influences reward‐ and motivation‐related aspects of food acquisition and consumption. While GLP‐1R agonist‐induced anorexigenic effects are mediated in part through hypothalamic and hindbrain circuits that regulate “homeostatic” processes influencing food intake, central GLP‐1R signaling also modulates non‐homeostatic hedonic‐based eating by reducing the reward value of palatable foods and the motivation to obtain them. These effects are achieved primarily through alterations in mesolimbic DA signaling (e.g., DA VTA input to the ACB), thus making GLP‐1R action particularly relevant to the study of overeating palatable foods.

Endogenous central GLP‐1 is produced primarily by preproglucagon (PPG)–expressing neurons in the nucleus tractus solitarius (NTS), which project directly to and release GLP‐1 in both the VTA and ACB (Alhadeff et al. 2012; Dossat et al. 2011, 2013). This hindbrain source of GLP‐1 provides a physiological brake on food reward, as blockade of endogenous GLP‐1R signaling with the GLP‐1R antagonist exendin 9–39 (Ex‐9) in the VTA or ACB significantly increases palatable high‐fat diet (HFD) consumption (Alhadeff et al. 2012; Dossat et al. 2011; Alhadeff et al. 2014), whereas chemogenetic activation of PPG neurons and their VTA projections produces the opposite effect (Wang et al. 2015). Together, these findings indicate that endogenous GLP‐1 released from hindbrain PPG neurons serves a physiological role in modulating mesolimbic reward processing, providing an intrinsic brake on food reward and motivation.

Pharmacological GLP‐1RAs also robustly engage central reward circuits to suppress hedonic feeding. Exogenously delivered GLP‐1RAs, whether given peripherally or intracerebroventricularly (ICV), act on GLP‐1Rs in reward‐related brain regions (Hernandez et al. 2018, 2019), and behavioral assays in rodents consistently demonstrate their effects on food reward. For example, in the progressive ratio (PR) operant lever‐pressing paradigm, first generation GLP‐1RAs (e.g., Ex‐4 and liraglutide) lower the break point, or maximal effort exerted to work for palatable foods (Dickson et al. 2012). Peripheral Ex‐4 also attenuates nose poke responses to incentive cues (ICs) predictive of sucrose (Wakabayashi et al. 2024), reduces the conditioned preference for palatable food rewards (Dickson et al. 2012), and diminishes unconditioned licking for sweet and fat solutions in brief‐access tests, suggesting a reduced hedonic valuation of palatable tastants (Treesukosol and Moran 2022). Newer and longer‐acting GLP‐1RAs such as semaglutide produce similar effects (Gabery et al. 2021; Ghidewon et al. 2022), and these effects also appear to extend to the next generation of non‐peptidergic small‐molecule GLP‐1RAs, including danuglipron and orforglipron (Godschall et al. 2024). Together, these converging behavioral findings demonstrate that pharmacological activation of GLP‐1Rs decreases both the reward value of food and the motivation to obtain it.

To pinpoint the underlying neuroanatomical substrates, site‐specific infusion studies have been performed. Intra‐VTA Ex‐4 administration markedly reduces HFD but not chow intake, indicating that VTA GLP‐1R signaling preferentially constrains palatable feeding (Alhadeff et al. 2012). VTA GLP‐1R activation also lowers motivation to work for palatable sucrose rewards (Dickson et al. 2012) and blockade of these receptors prevents systemic Ex‐4 from suppressing HFD intake (Mietlicki‐Baase et al. 2013). Similar effects are seen in the ACB, where GLP‐1R agonism decreases meal size and operant responding for palatable foods without producing aversive responses, whereas receptor blockade increases HFD consumption (Dossat et al. 2011, 2013). Beyond these canonical mesolimbic regions, GLP‐1R stimulation in the paraventricular thalamus (PVT) and lateral parabrachial nucleus (lPBN) also reduces palatable food intake and operant responding for palatable rewards (Alhadeff and Grill 2014; Ong et al. 2017). Selective GLP‐1R activation in the ventral hippocampus also potently reduces food consumption and palatable food‐motivated responses (e.g., progressive ratio operant test; food impulsivity measures) via downstream communication to the medial prefrontal cortex (mPFC) (Hsu et al. 2015, 2018). This suggests that hippocampal GLP‐1R signaling can curb the willingness to work for rewards, potentially by engaging connections between memory (e.g., hippocampus) and inhibitory control (e.g., mPFC) neural systems. Together, GLP‐1R signaling across mesolimbic nodes such as the VTA and ACB, as well as in interconnected regions like the PVT, lPBN, and hippocampus, is sufficient to suppress hedonic‐driven eating and food‐directed motivation. An interesting area for follow‐up investigation is to probe why these seemingly redundant anorexigenic GLP‐1‐responsive central nodes exist, given that activation of any independent node produces robust suppression of food‐directed behavior with minimal, if any, additive or synergistic effect from engaging additional nodes.

Building on these site‐specific infusion studies, recent work has shown that GLP‐1R activation suppresses food reward, at least in part, by dampening mesolimbic dopamine signaling (Konanur et al. 2020). While Ex‐4 crosses the blood–brain barrier (BBB) (Kastin and Akerstrom 2003), preliminary findings show that longer‐acting lipidated analogs like semaglutide may show less BBB penetration yet they still suppress VTA dopamine activity (Gabery et al. 2021; Abdulhameed et al. 2024; Zhu et al. 2025), potentially through indirect engagement of mesolimbic circuits via GLP‐1R–expressing regions in the hypothalamus and hindbrain that are accessible to circulating peptides, and/or through direct brain entry. Within the hindbrain, NTS GLP‐1R activation reduces high‐fat diet intake, lever pressing for sucrose under progressive ratio schedules, and conditioned place preference for palatable foods, while also increasing DA‐related gene expression in the VTA, including D2 receptor and tyrosine hydroxylase (Richard et al. 2015; Alhadeff and Grill 2014; Richard et al. 2015). Recent transcriptomics and RNAscope studies further revealed that VTA GLP‐1Rs are largely on inhibitory GABA‐ergic neurons, suggesting indirect modulation of local dopamine tone (Merkel et al. 2025). In the ACB, D2‐expressing medium spiny neurons, but not D1 neurons, mediate part of the feeding‐suppressive effects of GLP‐1R agonists (Sandoval‐Rodríguez et al. 2023).

In summary, endogenous GLP‐1 and its analogs suppress reward‐driven feeding in animal models by modulating, directly or indirectly, mesolimbic DA pathways that dampen the reinforcing properties of food and other rewards, but also through GLP‐1R action in distributed thalamic, hindbrain, and hippocampal circuitries. This highlights the therapeutic potential of targeting GLP‐1R pathways to address maladaptive eating behaviors and reward dysregulation, offering promising avenues for treating disorders such as obesity and binge eating.

#### 2.1.2 Substance Use Disorder

The influence of GLP‐1R activation on motivation and reward extends beyond food intake, as both drug reward and food reward processes rely on overlapping neural circuits and mechanisms within the mesolimbic and extended reward pathway (Koob and Volkow 2010). Over the past decade, this overlap has prompted growing interest in whether GLP‐1R agonists can modulate the rewarding effects of addictive substances. Early evidence emerged in the early 2010s, when pioneering work spearheaded by Elisabet Jerlhag and her team showed that systemic administration of Ex‐4 reduces alcohol, nicotine, amphetamine, and cocaine‐induced hyperactivity, ACB DA release, and conditioned place preference in mice (Egecioglu et al. 2013a, 2013b, 2012). Subsequent work extended these findings to both mice and rats across multiple addictive substances, including alcohol, psychostimulants, opioids, and nicotine, revealing both shared and substance‐specific roles of GLP‐1R signaling in addiction‐related behaviors.

##### 2.1.2.1 Alcohol

Among the various addictive substances examined, alcohol has received the most extensive investigation in the context of GLP‐1R signaling. Systemic administration studies consistently demonstrate that GLP‐1RAs attenuate alcohol intake and alcohol‐seeking behaviors across rodent and primate models. Early work with Ex‐4 and liraglutide showed decreases in voluntary drinking, operant self‐administration, and DA release for alcohol, while blockade of endogenous GLP‐1R signaling had the opposite effect, highlighting a physiological role in regulating alcohol reinforcement (Egecioglu et al. 2012; Shirazi et al. 2013; Sørensen et al. 2016; Suchankova et al. 2015). More recently, semaglutide has been shown to potently and dose‐dependently reduce voluntary, binge‐like, and dependence‐induced alcohol drinking in rodents (Aranäs et al. 2023; Chuong et al. 2023; Marty et al. 2020) and similar findings extend to non‐human primates (Thomsen et al. 2019). Importantly, preclinical studies indicate that prolonged treatment with GLP‐1R agonists can also prevent relapse‐like drinking. For example, daily Ex‐4 administration for 2 weeks attenuates deprivation‐induced rebound drinking without affecting locomotion or body weight (Thomsen et al. 2017), while short‐term repeated semaglutide administration suppresses alcohol intake and relapse‐like behavior in both sexes (Aranäs et al. 2023).

To delineate the neuroanatomical substrates, initial work in the VTA demonstrated that direct GLP‐1 or Ex‐4 infusion potently reduced alcohol intake (Shirazi et al. 2013). Extending beyond the VTA, intra‐ACB infusion of Ex‐4 suppresses alcohol‐induced locomotor stimulation and conditioned place preference in mice, and to reduce alcohol intake in rats with a history of heavy consumption, implicating mesolimbic GLP‐1Rs as central regulators of alcohol reinforcement (Colvin et al. 2020; Vallöf, Kalafateli, and Jerlhag 2019). In the hindbrain, intra‐NTS Ex‐4 reduced alcohol consumption, blunted alcohol‐induced dopamine release in the ACB, and disrupted reward memory consolidation, pointing to a strong influence of ascending GLP‐1R signaling pathways (Vallöf, Vestlund, and Jerlhag 2019). More recently, hippocampal and septal circuits have been implicated, as Ex‐4 infusions into the dorsomedial hippocampus (dHPC), ventral hippocampus (vHPC), or Lateral Septum (LS) robustly suppressed alcohol self‐administration at doses comparable to systemic administration (Colvin et al. 2020; Allingbjerg et al. 2023). In particular, LS GLP‐1R activation attenuated alcohol's rewarding effects by reducing locomotor stimulation, place preference, and accumbal dopamine release, while inhibition of LS GLP‐1Rs produced the opposite effect (Edvardsson et al. 2025).

Although most studies have focused on males, evidence points to important sex‐specific differences in GLP‐1R regulation of alcohol behaviors. Female rodents generally consume more alcohol than males (Priddy et al. 2017), and in female rats, Ex‐4 reduced intake when delivered into the ACB shell but not the core, suggesting striatal region‐selective sensitivity (Abtahi et al. 2018). In male mice, systemic Ex‐4 administration suppressed both reinstatement of alcohol seeking and alcohol self‐administration, whereas females showed little response (Díaz‐Megido and Thomsen 2023). Long‐term treatment with dulaglutide reduced ethanol intake and preference in both sexes, but the effect persisted after discontinuation only in males (Vallöf et al. 2020). Taken together, these findings suggest that while GLP‐1R agonists attenuate alcohol‐related behaviors in both sexes, the magnitude, persistence, and neural locus of these effects may differ, underscoring the need to include sex as a biological variable in future studies.

Mechanistic studies are beginning to clarify how GLP‐1R agonists act within reward circuits to suppress alcohol‐related behaviors. Fluorescently labeled semaglutide has been detected in the ACB shell of both male and female alcohol‐drinking rats, suggesting that blood–brain barrier penetration may be a mechanism to allow semaglutide to act locally within mesolimbic circuits (Aranäs et al. 2023). Semaglutide also increased sIPSC frequency in CeA and ILC neurons of alcohol‐naïve rats, suggesting an enhancement of GABA release, though this effect was blunted in alcohol‐dependent animals (Chuong et al. 2023). Similarly, LS GLP‐1R expression correlated with alcohol intake in males, and receptor activation reduced alcohol reward by depressing local GABAergic neurotransmission in a GABA_A_‐dependent manner (Edvardsson et al. 2025). Collectively, these findings point to a convergence of mechanisms in which GLP‐1R signaling dampens alcohol's reinforcing properties by both limiting mesolimbic dopamine activity and enhancing inhibitory GABAergic tone.

##### 2.1.2.2 Psychostimulants

Although alcohol represents the most extensively studied and supported case, emerging evidence also implicates GLP‐1R signaling in the modulation of psychostimulant reward, particularly cocaine and amphetamine. Systemic administration of Ex‐4 reduces both acute and chronic cocaine self‐administration, hyperlocomotion, and blunts increases in striatal DA and the expression of the marker of neuronal c‐fos (Egecioglu et al. 2013b; Sørensen et al. 2015). In addition, systemic Ex‐4 attenuates amphetamine‐induced hyperactivity, reduces ACB DA release, and suppresses conditioned place preference in mice (Egecioglu et al. 2013b). Conversely, GLP‐1R‐deficient mice exhibit exaggerated cocaine‐induced locomotor responses and conditioned place preference compared with wild‐type littermates (Harasta et al. 2015). Notably, Ex‐4 failed to attenuate amphetamine‐induced conditioned place preference in animals lacking CNS GLP‐1Rs, suggesting that this behavioral effect is mediated primarily through central GLP‐1R populations (Sirohi et al. 2016). Site‐specific studies implicate multiple reward‐related regions: intra‐VTA and intra‐ACB Ex‐4 infusions suppress cocaine seeking and reinstatement, while NTS‐to‐LDTg pathways attenuate drug‐seeking through GLP‐1R‐dependent mechanisms (Hernandez et al. 2018, 2019, 2021). Mechanistically, GLP‐1R activation dampens psychostimulants' reinforcing properties by attenuating phasic DA signaling within the mesolimbic VTA–ACB pathway, with VTA GLP‐1Rs emerging as a key substrate engaged by ascending hindbrain inputs (Merkel et al. 2025; Sørensen et al. 2015; Schmidt et al. 2016; Fortin and Roitman 2017).

##### 2.1.2.3 Opioids

Compared to alcohol and psychostimulants, the role of GLP‐1R signaling in opioid reinforcement is less studied and appears more complex. Systemic Ex‐4 binds to putative GLP‐1Rs located on both D1‐ and D2‐expressing medium spiny neurons in the ACB shell, directly modulating striatal output (Zhang et al. 2020). Both systemic and intra‐ACB shell Ex‐4 reduce oxycodone self‐administration and reinstatement in rats (Zhang et al. 2020). Importantly, Ex‐4 did not alter the analgesic effects of oxycodone, suggesting that activation of GLP‐1Rs may attenuate opioid reinforcement without compromising analgesia (Zhang et al. 2020). Similarly, Ex‐4 reduces cue‐induced and drug‐induced reinstatement of heroin seeking (Douton et al. 2021), and both acute and chronic liraglutide treatment delay heroin initiation, decrease intake, and prevent reinstatement (Douton, Horvath, et al. 2022; Douton, Acharya, et al. 2022). More recent studies extend these findings to synthetic opioids. Acute liraglutide treatment attenuates both cue‐induced and drug‐induced reinstatement of fentanyl seeking in both male and female rats, with particularly pronounced effects observed during estrus in females (Urbanik et al. 2022, 2025). Together, these findings suggest that GLP‐1R agonists can reduce relapse‐like behaviors for several opioids; however, not all opioids appear equally sensitive to GLP‐1R modulation (Bornebusch et al. 2019), suggesting that the efficacy of GLP‐1RAs may vary across compounds, species, and experimental paradigms.

##### 2.1.2.4 Nicotine

Systemic administration of GLP‐1RAs reduces nicotine intake, relapse‐like behaviors, and withdrawal‐associated hyperphagia. Ex‐4 decreases nicotine self‐administration, while liraglutide suppresses nicotine‐evoked DA release in the ACB and attenuates nicotine self‐administration, extinction, reinstatement in both sexes, and importantly, withdrawal‐associated hyperphagia and weight gain (Falk et al. 2023; Herman et al. 2023; Tuesta et al. 2017). Mechanistic studies reveal that nicotine activates PPG neurons in the NTS, which project to the MHb–IPN circuit. GLP‐1R activation within the MHb–IPN pathway suppresses nicotine reward and operant intake, supporting the idea that this circuit functions as a “satiety sensor” promoting nicotine avoidance (Tuesta et al. 2017). The engagement of this pathway represents a mechanistically distinct mode of GLP‐1R action compared to the mesolimbic VTA–ACB reward circuitry implicated in food and other addictive drugs. Complementary work shows that liraglutide also dampens nicotine‐induced dopamine release in the ACB (Falk et al. 2023), underscoring the convergence of GLP‐1R actions on both mesolimbic dopamine signaling and habenular pathways. Together, these findings indicate that systemic GLP‐1R agonists suppress nicotine reinforcement by engaging hindbrain GLP‐1 neurons, modulating the MHb–IPN avoidance circuit, and reducing mesolimbic dopamine output.

##### 2.1.2.5 Converging Mechanisms

Collectively, work across palatable food, alcohol, psychostimulants, opioids, and nicotine demonstrates that GLP‐1R activation attenuates drug reward through action in mesolimbic, hindbrain, hippocampal, and septal circuitry. A recurring mechanism that emerges across these substances involves the recruitment of GABAergic neuronal populations within the VTA, leading to increased inhibitory tone onto dopaminergic neurons and consequent dampening of DA signaling, thereby reducing both the hedonic impact of drugs and the motivational salience of drug‐associated cues. Collectively, evidence from animal models position GLP‐1R signaling as a convergent modulator of reinforcement and relapse‐like behavior, although the precise neuronal populations and circuit‐level pathways remain to be completely defined. While future work is needed to determine whether separate central populations of GLP‐1Rs (or GLP‐1‐based drugs) can suppress motivation for distinct rewards (e.g., food vs. opioid vs. alcohol), the literature thus far paints a picture of a high degree of overlap between neural target systems that mediate consumption of palatable foods and various addictive substances.

### 2.2 Clinical Evidence in Humans

#### 2.2.1 Food Intake

The ability of GLP‐1RAs to influence food‐related reward and motivation emerged shortly after GLP‐1 analogs such as exenatide and liraglutide began to be widely used for the management of obesity and T2DM management. Across multiple human randomized controlled trials, GLP‐1R agonist treatment reduces energy intake, food cravings, and preference for highly palatable, energy‐dense foods, while improving subjective control over eating behavior (Blundell et al. 2017; Inoue et al. 2011). Importantly, these effects are observed in the absence of marked reductions in food liking, suggesting a primary influence on motivational drive rather than consummatory pleasure (Coppin et al. 2023).

Consistent with these behavioral effects, neuroimaging studies demonstrate that obese individuals, including those with T2DM, exhibit heightened neural responses to food cues in appetite‐ and reward‐related regions compared with lean participants, and that treatment with GLP‐1R agonists normalizes this exaggerated cue reactivity (Kulve et al. 2016; van Bloemendaal et al. 2014). For example, acute administration of Ex‐4 reduces food intake and dampens activation in the insula, amygdala, putamen, and orbitofrontal cortex, effects that are blocked by GLP‐1R antagonism (van Bloemendaal et al. 2014). In line with these findings, shorter‐term liraglutide treatment reduces neural responses to highly desirable food cues in reward‐ and attention‐related regions, including the parietal cortex, insula, and putamen, with cue‐evoked brain activity correlating with subjective ratings of hunger and appetite (Farr et al. 2016). Similar modulation of neural responses to food cues and taste stimuli has also been reported with semaglutide treatment (Jensterle et al. 2025). However, not all studies point to sustained neural suppression, as longer‐term liraglutide treatment may elicit compensatory increases in orbitofrontal cortex activation when controlling for weight loss, potentially contributing to plateaus in treatment efficacy over time (Farr et al. 2019). Collectively, these findings indicate that GLP‐1RAs reduce craving and incentive salience for highly palatable foods while largely sparing consummatory hedonic responses.

#### 2.2.2 Substance Use Disorder

##### 2.2.2.1 Alcohol

From clinical evidence in food reward and early anecdotal reports of reduced alcohol and drug cravings among patients prescribed GLP‐1RAs for metabolic indications, this prompted targeted investigation into their potential utility for substance use disorders. Most clinical studies to date have examined alcohol and nicotine, though trials investigating other addictive drugs are currently ongoing (ClinicalTrials.gov identifiers: NCT07227948, NCT06548490, NCT04199728, NCT06651177). In Alcohol Use Disorder (AUD) patients, once‐weekly exenatide reduces fMRI alcohol cue reactivity in the ventral striatum and septal area and decreases heavy drinking days, particularly in obese patients (Klausen et al. 2022). Similarly, dulaglutide significantly reduces alcohol consumption relative to placebo, and low doses of semaglutide reduce laboratory alcohol self‐administration, drinks consumed per drinking day, and weekly alcohol cravings (Hendershot et al. 2025; Probst et al. 2023). Real‐world and observational studies further support these findings, reporting reduced alcohol consumption among patients treated with liraglutide or semaglutide, as well as lower self‐reported alcohol use and Alcohol Use Disorders Identification Test (AUDIT) scores among individuals taking semaglutide or tirzepatide (Quddos et al. 2023).

##### 2.2.2.2 Nicotine

In contrast to alcohol, clinical evidence for nicotine use disorder (NUD) has consistently demonstrated metabolic rather than behavioral benefits. Across studies, GLP‐1RAs consistently reduce nicotine withdrawal‐induced body weight gain, representing the most robust and reproducible clinical finding across studies. Both extended release exenatide combined with nicotine replacement therapy (NRT) (Yammine et al. 2023) and dulaglutide combined with varenicline (Lengsfeld et al. 2023) significantly attenuated post‐cessation weight gain compared to standard smoking cessation treatments alone. In contrast, evidence supporting a direct effect of GLP‐1R agonists on smoking cessation outcomes is mixed and context‐dependent. Extended release exenatide administered alongside NRT increased short‐term smoking abstinence, reduced cigarette craving, and alleviated withdrawal symptoms, but these effects were observed specifically in participants who achieved abstinence (Yammine et al. 2023). Conversely, no additional benefit of dulaglutide was detected when combined with varenicline with respect to abstinence rates or craving (Lengsfeld et al. 2023). Collectively, current evidence supports GLP‐1R agonists as promising adjunctive therapies rather than stand‐alone treatments for NUD. To date, no published clinical trial has evaluated GLP‐1R agonist monotherapy for smoking cessation. However, ongoing and recently completed trials are beginning to address this gap (ClinicalTrials.gov identifier: NCT03712098, NCT05530577). Notably, the two completed trials to date were of relatively short duration, limiting conclusions about long‐term efficacy. Ongoing and planned studies incorporating longer treatment and follow‐up periods, as well as combination approaches pairing GLP‐1RAs with counseling and NRT, will be critical to determine the durability of the effects on post‐cessation weight gain, abstinence, and relapse risk (ClinicalTrials.gov identifiers: NCT05610800, NCT06173778; see (Herman and Schmidt 2024) for review).

##### 2.2.2.3 Psychostimulants

Evidence for GLP‐1RA efficacy in cocaine use disorder (CUD) remains limited but is rapidly evolving. While preclinical rodent studies have demonstrated that GLP‐1As reduce cocaine intake, acute pretreatment with a low dose of exenatide did not affect cocaine self‐administration or the subjective effects of cocaine in individuals with CUD in a randomized controlled trial (Angarita et al. 2021). However, the surprising lack of effect in the cocaine trial, along with study limitations like the use of a single acute dose, prevents firm conclusions. Supporting the possibility that sustained GLP‐1RA treatment may be required to observe clinical effects, a recent case report described a patient with comorbid obesity and cocaine use disorder who exhibited marked reductions in cocaine craving alongside significant weight loss following 12 weeks of semaglutide treatment (Romeo 2025). Importantly, several ongoing and recently completed trials are now evaluating sub‐chronic and chronic GLP‐1RA treatment in both treatment‐seeking and non–treatment‐seeking individuals with CUD (ClinicalTrials.gov identifiers: NCT02302976, NCT07227948, NCT06691243).

##### 2.2.2.4 Opioid

Ongoing clinical trials are evaluating GLP‐1RAs for opioid use disorder (OUD) using sustained, multi‐week treatment paradigms. These studies include GLP‐1RA monotherapy as well as adjunctive GLP‐1RA and tirzepatide treatments alongside opioid therapies (ClinicalTrials.gov identifiers: NCT04199728, NCT06548490, NCT06639464, NCT06651177). Although most available trials remain small and many are still ongoing, their emergence reflects growing interest in repurposing long‐acting GLP‐1 analogs for addiction treatment across substances.

Collectively, these preclinical and clinical studies suggest that GLP‐1R signaling regulates not only food reward and motivation but also neural circuits underlying addiction to addictive drugs. By attenuating the rewarding effects of these drugs, in part by modulating mesolimbic dopaminergic activity, GLP‐1R activation offers a promising therapeutic avenue for the future treatment of substance use disorder. The cumulative results of recent, ongoing, and near‐future clinical trials will soon determine the clinical efficacy of GLP‐1RAs for addiction.

## 3 Affective and Anxiety Disorders

### 3.1 Preclinical Evidence

Affective disorders (i.e., mood disorders) are a set of psychiatric conditions that are marked by abnormal emotional states that negatively affect quality of life and general well‐being. Two main types of affective disorders are depression (Spijker and Claes 2014; Psychiatry 2013), characterized by prolonged periods of sadness and a diminished enjoyment of previously enjoyable activities, and bipolar disorder, which involves significant mood state fluctuations between manic (extreme happiness and heightened mood) and depressive episodes (Sekhon and Gupta 2025). Anxiety disorders represent a related and prevalent class of psychiatric conditions characterized by excessive fear and/or nervousness about real and/or perceived threats. Anxiety disorders frequently co‐occur with affective disorders and share similar symptoms, a comorbidity that further disrupts daily activities and negatively impacts quality of life.

As discussed in the previous section, GLP‐1 and its receptor agonists modulate reward and motivation, at least in part, by dampening dopamine signaling. Given these effects and their profound impact on food intake and weight loss, a broad range of research has examined whether GLP‐1RAs also influence mood‐related outcomes such as anxiety, depression, and anhedonia. While modeling affective disorders in rodents is inherently complex and comes with limitations, several validated behavioral assays allow researchers to assess physiological and behavioral responses that serve as meaningful analogs to human emotional states. Here, we explore how GLP‐1R activation affects anxiety‐ and depression‐like behaviors in rodent models using established behavioral assays. Anxiety‐like behaviors are commonly assessed with the elevated plus maze (EPM) and open field test (OFT), where decreased time spent in open or unprotected areas indicates an anxiety phenotype. Depression‐like behaviors are evaluated using the forced swim test (FST) and tail suspension test (TST), where passive coping strategy, such as immobility, is interpreted as behavioral despair.

In experimental rodent models, emerging evidence suggests that GLP‐1RAs can exert complex and sometimes bidirectional effects on affective states, which largely depend on drug treatment duration rather than differences between specific GLP‐1RAs. For example, acute systemic as well as lateral ventricle infusion of Ex‐4 in male rats increases anxiety‐like behaviors in the EPM and OFT by reducing time spent in open areas (Anderberg et al. 2016). Similarly, systemic injection of liraglutide acutely increases anxiety‐like behavior in the OFT and EPM in both mice and rats (Kamble et al. 2016). Consistent with these findings, acute systemic administration of Ex‐4 elevates anxiety‐like behaviors in the OFT and promotes behavioral despair by increasing immobility time in the forced swim test (FST) in the WAG/Rij rat, a rat model exhibiting absence epilepsy and depressive‐like behaviors (Aygun 2021).

On the contrary, chronic administration of GLP‐1RAs reduces anxiety and depressive‐like behaviors in various rodent models of depression. For instance, in mouse models of chronic unpredictable stress (CUS), daily systemic injections of liraglutide significantly reduce behavioral despair in the FST (Seo et al. 2023). Notably, chronic liraglutide treatment also exerts similar antidepressant‐like effects in naïve mice that did not undergo the CUS paradigm (Seo et al. 2023). Likewise, twice‐weekly systemic dulaglutide injections or daily intranasal treatment of lixisenatide over 3 weeks in CUS‐exposed mice lead to significant reductions in behavioral despair in the FST and TST, as well as reduced anxiety‐like behavior in the OFT (Jin et al. 2024) and EPM (Ren et al. 2021). These findings are consistent across multiple rodent depression models, as chronic systemic liraglutide administration also yields antidepressant and anxiolytic effects in models of depression induced by Corneal/PTZ Kindling Epilepsy (Koshal and Kumar 2016a, 2016b), corticosterone (Weina et al. 2018), olanzapine (Sharma et al. 2015), and lipopolysaccharide (Ventorp et al. 2017). Additionally, once‐weekly systemic dulaglutide injections over 4 weeks restore sucrose preference in male mice exposed to chronic social defeat stress, indicating a potential reduction of anhedonia (Darwish et al. 2023). Importantly, these antidepressant‐like effects were not secondary to weight loss or hypophagia, as pair‐fed control rats failed to show mood improvements following chronic central administration of Ex‐4 in the FST (Anderberg et al. 2016).

Given the comorbidity between affective and anxiety disorders and metabolic impairments (Anderson et al. 2001; Fulton et al. 2022), an important area of research relates to the efficacy of GLP‐1RAs in alleviating anxiety and depression‐like behaviors associated with obesity and/or metabolic disorders. Twice daily systemic injections of Ex‐4 for 2 weeks decrease behavioral despair in the FST in a streptozocin‐ and nicotinamide‐induced mouse model of diabetes. The same treatment also reduces anxiety‐like behaviors in the EPM (Komsuoglu Celikyurt et al. 2014). Similarly, in the *db/db* leptin receptor mutation diabetic mouse model, once daily systemic administration of Ex‐4 for 4 days exhibits anxiolytic and antidepressant effects in anxiety and depression‐like behaviors in OPT, EPM, FST, and TST (Yang et al. 2022). In mice maintained on a high‐fat diet that promotes weight gain and impairs glucose metabolism, once‐weekly systemic injections of semaglutide for 6 weeks reduce anxiety and depression‐like behaviors associated with obesity in the EPM, TST, and FST (de Paiva et al. 2024).

Stress is a critical factor in the development and exacerbation of anxiety disorders. One mechanism through which acute GLP‐1R activation contributes to anxiety involves modulation of the hypothalamic–pituitary‐adrenocortical (HPA) axis. This pathway, centrally initiated by corticotropin‐releasing hormone (CRH)‐expressing neurons in the paraventricular nucleus (PVN) of the hypothalamus (Herman et al. 2016), is influenced by GLP‐1 signaling. Intracerebroventricular infusion of GLP‐1 activates PVN CRH neurons (Gil‐Lozano et al. 2014; Larsen et al. 1997), elevates adrenocorticotropic hormone (ACTH) and corticosterone (CORT) levels in rats (Larsen et al. 1997; Gil‐Lozano et al. 2010; Kinzig et al. 2003), and increases anxiety‐like behavior in the elevated plus maze (Kinzig et al. 2003). Similar effects are seen following systemic administration of Ex‐4, whereas pharmacological blockade of central GLP‐1R signaling reduces anxiety and dampens stress‐induced rises in ACTH and CORT (Kinzig et al. 2003). Anatomical evidence supports this functional relationship, as NTS PPG‐neurons form synaptic contacts with CRH neurons in the PVN (Sarkar et al. 2003), supporting a direct role of GLP‐1R signaling in the modulation of central stress responses. Translationally, GLP‐1R activation has also been linked to increased cortisol secretion in humans, indicating a conservation of this stress‐related pathway across species (Gil‐Lozano et al. 2010).

In contrast, the mechanisms underlying the chronic antidepressant and anxiolytic effects of GLP‐1RAs remain less well defined. These longer‐term benefits may reflect secondary improvements in energy metabolism, weight, and overall health, given that many chronic studies are conducted in disease‐relevant animal models characterized by systemic inflammation and metabolic dysregulation. Beyond metabolic improvements, GLP‐1RAs also exert neuroprotective and anti‐inflammatory effects and stabilize the gut–brain axis and microbiota, factors that may independently support mood regulation (Detka and Głombik 2021). Taken together, evidence suggests that while acute GLP‐1R activation directly enhances stress responses and anxiety, prolonged administration produces indirect antidepressant and anxiolytic effects across preclinical models. Future work is needed to disentangle whether these chronic benefits primarily arise from improved energy metabolism and/or reflect direct central mechanisms of GLP‐1R signaling, a distinction critical for fully harnessing their therapeutic potential in affective and anxiety disorders.

### 3.2 Clinical Evidence in Humans

Given that some weight loss drug targets have been associated with an increased risk of suicide, depression, and self‐harm (Mitchell et al. 2013), research on GLP‐1RAs has expanded in clinical studies to explore their impacts on overall quality of life, suicidal ideation, anxiety, and depression. While causal directionality is unclear, obesity and T2DM are often associated with a reduction in health‐related quality of life (HRQoL), which the FDA characterizes as a patient's perception of physical, psychological, and social well‐being impacted by illness and its treatment (Patrick et al. 2007). Numerous studies have shown the positive impact of GLP‐1RAs on HRQoL (Kolotkin et al. 2016, 2018; Jódar et al. 2020). For example, in the randomized SCALE Obesity and Prediabetes trial, liraglutide was associated with significant improvements in patient‐reported HRQoL after 1 year compared to placebo (Kolotkin et al. 2016), with benefits persisting over 3 years of once‐daily continued treatment (Kolotkin et al. 2018). Additionally, a post hoc analysis of data from 4725 participants across three randomized, placebo‐controlled, double‐blind trials found that 1 year of liraglutide treatment significantly improves HRQoL scores as measured by the Impact of Weight on Quality of Life–Lite (IWQoL‐Lite) scale (Bays et al. 2017).

Notably, some weight loss medications (e.g., the cannabinoid receptor type‐1 antagonist Rimonabant) have been associated with a potential increased risk of suicide, depression, and self‐harm (Akbas et al. 2009). In light of this, clinical research on GLP‐1RAs has increasingly focused on their impact on mental health outcomes. In 2023, the European Medicines Agency (EMA), the Food and Drug Administration (FDA), and the United Kingdom's Medicines and Healthcare Products Regulatory Agency (MHRA) launched investigations to evaluate the safety of GLP‐1RAs, given the established association between obesity and depressed mood, which can elevate suicidal risk (Heneghan et al. 2012). Importantly, most studies report no difference in suicidal ideation or behavior between GLP‐1RAs and other diabetes treatments (De Giorgi et al. 2024; Hurtado et al. 2024; Ueda et al. 2024; Nassar et al. 2024; Tagliapietra et al. 2024) using national databases such as FDA Adverse Event Reporting System (FAERS) (Chen et al. 2024; McIntyre et al. 2025) and Veterans Health Administration data (Tagliapietra et al. 2024). In a recent retrospective study using real‐world data from the global TriNetX health research database, individuals with T2DM treated with GLP‐1RAs consistently demonstrate a lower risk of suicide attempts compared to those receiving DPP‐4 inhibitors. This difference is particularly noteworthy among individuals with a history of depression or previous suicide attempts (Nassar et al. 2024), and may be driven by the far more potent weight loss effects in GLP‐1RAs compared to DPP‐4 inhibitors, as well as its intrinsic actions within the CNS. This is supported by a 16‐week randomized fMRI study of 30 women with obesity and polycystic ovary syndrome. Semaglutide treatment increases resting‐state functional connectivity (RSFC) in brain regions associated with suicidal ideation (Verovnik and Vovk 2024), which are regions previously shown to exhibit reduced RSFC in patients with major depressive disorder (Cao et al. 2020; Schmaal et al. 2020). In conclusion, current analyses do not support concerns of an increased risk of suicidal ideation with GLP‐1 analogs; on the contrary, some findings suggest that GLP‐1 analogs may be associated with a lower risk of suicidal ideation compared to other anti‐obesity and anti‐diabetes medications. The extent that these potentially beneficial effects are secondary to weight loss remains to be deeper investigated.

While chronic treatment with GLP‐1 analogs may have some beneficial effects on suicidal ideation, findings from clinical studies on the effects of GLP‐1RAs on affective and anxiety disorders remain mixed. For instance, a recent analysis indicated a 3‐fold increased risk of major depression and a 2‐fold increased risk of anxiety among GLP‐1RA users from 6 months to 5 years, suggesting that long‐term use may elevate the risk of these psychiatric conditions (Kornelius et al. 2024). On the contrary, another recent meta‐analysis of randomized controlled trials with GLP‐1 analog treatments of at least 52 weeks found no adverse effects on any mental health outcomes (Silverii et al. 2024). Consistent with these findings, a population‐based cohort study using the UK Clinical Practice Research Datalink (CPRD) from January 2007 to January 2016 found no association between GLP‐1‐based therapies and an increased or decreased incidence of depression when compared to sulfonylureas, which is one of the most commonly prescribed drugs for the management of T2DM worldwide (Gamble et al. 2018). Similarly, no association has been found between liraglutide or semaglutide use and an increased risk of depression relative to DPP‐4 inhibitor treatments (Tagliapietra et al. 2024). In other studies, GLP‐1RAs are found to have beneficial effects on affective disorders. A 2024 analysis examined data from a large and diverse population of 3,081,254 diabetic patients and 929,174 non‐diabetic patients, concluding that diabetic patients prescribed tirzepatide and semaglutide were on average 50% less likely to be diagnosed with depression and anxiety post treatment compared to non‐users (Miller et al. 2024). Similarly, several other studies have also observed antidepressant and anxiolytic effects of GLP‐1RAs (Chen et al. 2024; Moulton et al. 2016; Tsai et al. 2022; Wium‐Andersen et al. 2022). A meta‐analysis found GLP‐1RAs to outperform control treatments in reducing depression scores, analyzing data from both depressed and non‐depressed patients with diabetes (Pozzi et al. 2019). Additionally, a 4‐week open‐label trial in 19 non‐diabetic patients with MDD or BD and below‐average cognitive performance reported a significant reduction in depressive symptoms based on the Hamilton Depression Rating Scale (HAM‐D) by the end of the 4‐week daily injections of liraglutide treatment compared to baseline (Mansur et al. 2017).

The differing outcomes across studies may reflect variations in sample populations, with studies including individuals with major depressive disorder (MDD), prior suicidal ideation, and longer follow‐up periods yielding distinct findings. This variability underscores the need for further research to clarify the complex effects of GLP‐1RAs on affective and anxiety disorders. Overall, however, treatment with GLP‐1RAs for diabetes or weight loss does not appear to present a risk for elevated affective and anxiety disorders. Given that GLP‐1RAs are now widely accessible to (and used by) large populations of individuals who are not diabetic or obese, it is important for future research to evaluate the impact of these drugs on affective and anxiety disorders in these populations as well.

## 4 Memory and Learning

### 4.1 Preclinical Evidence

Learning and memory impairments encompass a spectrum of cognitive disorders characterized by difficulties in acquiring, retaining, or retrieving information, with significant negative effects on daily functioning and quality of life. These impairments manifest in several ways: as difficulties forming new memories (anterograde amnesia), recalling past information (forgetfulness, retrograde amnesia), or acquiring cognitive skills despite adequate intelligence, training, and education. Mental decline from aging and neurodegenerative diseases represents another major category, ranging from mild cognitive impairment to severe dementia, including Alzheimer's Disease (AD) (Porsteinsson et al. 2021).

Beyond genetic susceptibility, metabolic disorders, particularly diabetes and obesity, are causally connected to learning and memory impairments (Tsan et al. 2021; Srikanth et al. 2020). T2DM doubles the risk of developing dementia and accelerates cognitive aging (Antal et al. 2022), while obesity is linked to deficits in executive function, working memory, and episodic memory (Smith et al. 2011). Mechanistically, the metabolic dysregulation associated with these conditions promotes neuroinflammation and oxidative stress, which broadly disrupt distributed neuronal circuits involved in cognition, including cortical, limbic, and hippocampal networks. Among these, hippocampal processes have been particularly well studied, with growing evidence suggesting that metabolic stress disrupts hippocampal synaptic plasticity and adult neurogenesis, both of which are essential for memory formation and consolidation (Lazarov et al. 2020).

Accumulating preclinical evidence suggests that GLP‐1RAs exert neuroprotective and cognitive‐enhancing effects, highlighting their potential in counteracting learning and memory impairments. Whether these effects are mediated by direct receptor action in brain memory systems (e.g., hippocampus), indirect benefits from improved glucose metabolism and weight loss, or a combination of both remains an active area of investigation. A growing body of preclinical studies now provides convergent evidence that GLP‐1R signaling influences learning and memory across multiple biological levels, ranging from neuronal survival and growth to synaptic plasticity and behavior.

GLP‐1 exerts neuroprotective effects through multiple neurobiological mechanisms at the cellular level. In cultured cells, native GLP‐1 peptide induces neurite outgrowth in a manner similar to the neurotrophin nerve growth factor (NGF) (Perry, Lahiri, et al. 2002). Both GLP‐1 and Ex‐4 protect cultured rat hippocampal neurons against glutamate‐induced apoptosis and reduce hippocampal neuroinflammation in vivo (Cui et al. 2020; Perry, Haughey, et al. 2002). These findings identify a novel neuroprotective and neurotrophic function of hippocampal GLP‐1R signaling. GLP‐1RAs also enhance synaptic structure and function by promoting hippocampal long‐term potentiation (LTP), a core mechanism of memory encoding, across both physiological and pathological conditions. Ex‐4 restores impaired LTP and dendritic spine maturation in an in vitro model for metabolic dysfunction, partly through improved insulin signaling and synaptic protein expression (Wang et al. 2021). Liraglutide similarly enhances hippocampal LTP when administered centrally, an effect abolished by GLP‐1R antagonism, confirming direct receptor‐mediated action on hippocampal circuits (McClean et al. 2010). Notably, central administration of Ex‐9 impaired LTP, suggesting that endogenous GLP‐1 tone may contribute to basal hippocampal synaptic plasticity. In contrast, evidence linking GLP‐1R signaling to long‐term depression (LTD) is limited; to our knowledge, no studies have directly demonstrated GLP‐1‐dependent LTD induction, though indirect modulation of inhibitory tone and synaptic homeostasis has been reported in hippocampal CA3 slices (Korol et al. 2015). Thus, while GLP‐1RAs reliably promote LTP and synaptic strengthening, their role in LTD remains unclear.

In rodent models of Alzheimer's Disease (AD), GLP‐1RAs improve learning and memory while reducing pathological hallmarks. For example, liraglutide treatment reduces amyloid beta (Aβ) deposition and phosphorylated tau levels in various rodent AD models, suggesting neuroprotective effects against AD pathology (Qi et al. 2016; Hansen et al. 2015). Beyond disease models, liraglutide enhances learning and memory in wildtype rodents. For example, liraglutide treatment mitigated early‐life hippocampal neuroinflammation caused by perinatal maternal food restriction by reducing neuroinflammatory signaling and glial activation in offspring (Diz‐Chaves et al. 2018).

Genetic studies further support GLP‐1R's role in cognition. For example, acute activation of brain GLP‐1 receptors enhances associative and spatial learning, whereas genetic loss of GLP‐1R leads to learning deficits that are rescued by restoring receptor expression in the hippocampus. Extending these findings, hippocampal GLP‐1R overexpression further improves learning and memory, while GLP‐1R‐deficient mice exhibit enhanced susceptibility to kainate‐induced seizures and hippocampal neuronal injury, with heterozygotes displaying an intermediate phenotype (During et al. 2003). Phenotypic correction occurs after GLP‐1R gene transfer into hippocampal somatic cells, reinforcing that brain GLP‐1R represents a promising target for both cognitive‐enhancing and neuroprotective agents.

Dual GLP‐1/GIP receptor agonists have also demonstrated potent neuroprotective actions in AD mouse models, even surpassing effects of GLP‐1RA monotherapies. The dual agonist DA4‐JC robustly improves memory and hippocampal LTP while reducing amyloid pathology and neuroinflammation in APP/PS1 mice, outperforming liraglutide (Maskery et al. 2020). In APP/PS1 and other transgenic AD models, dual agonists markedly improve spatial and recognition memory and restore hippocampal LTP. They also increase dendritic spine and synapse density, elevate the synaptic proteins PSD‐95 and synaptophysin, and reduce amyloid‐β burden, tau hyperphosphorylation, neuroinflammation, and mitochondrial dysfunction (Cai et al. 2021). Together, these results demonstrate that GLP‐1/GIP co‐agonism corrects synaptic deficits and reduces AD‐related pathology, positioning dual‐incretin agonists as compelling candidates for Alzheimer's therapeutics.

### 4.2 Clinical Evidence in Humans

T2DM and obesity are well‐established risk factors for cognitive decline and dementia in humans. Large meta‐analytic and cohort studies show that T2DM increases dementia risk by approximately 50%–60%, with diabetic adults exhibiting accelerated global cognitive decline compared to non‐diabetic individuals (Biessels et al. 2014). Midlife obesity similarly confers a 41% higher risk of dementia, independent of vascular comorbidities, and the combination of obesity with early‐onset T2DM predicts the highest dementia hazard (Qi et al. 2024; Pedditzi et al. 2016). These associations likely reflect converging metabolic, inflammatory, and vascular mechanisms, including insulin resistance, chronic low‐grade inflammation, cerebrovascular impairment, and disrupted neuronal energy metabolism. Together, these processes accelerate synaptic vulnerabilities, providing a rationale for evaluating GLP‐1‐based therapies in cognitive decline and Alzheimer's disease (Lemche et al. 2024).

Recent population‐based analyses have consistently shown that GLP‐1RAs are associated with reduced risk of cognitive decline and dementia. A 2025 study using over 2 million adults from U.S. Veterans Affairs databases found that GLP‐1RA exposure was associated with significantly lower incidence of Alzheimer's disease and all‐cause dementia compared with DPP‐4 inhibitors or sulfonylureas, independent of glycemic control or cardiovascular risk factors (Xie et al. 2025). Similarly, a nationwide U.S. insurance‐claims analysis reported that adults initiating GLP‐1RAs had significantly lower risk of developing dementia relative to those prescribed DPP‐4 inhibitors (Seminer et al. 2025). These findings suggest that GLP‐1RAs may confer neuroprotective benefits even outside formal clinical trial settings, possibly through synergistic metabolic and anti‐inflammatory pathways.

Multiple neuroimaging trials have demonstrated that GLP‐1RA treatment modulates brain metabolism and neural network function in humans. In a randomized crossover FDG‐PET study, exenatide increased cerebral glucose metabolic rate in brain regions related to glucose homeostasis, even with lower circulating insulin, indicating that GLP‐1RAs can modulate brain glucose utilization directly (Daniele et al. 2015). In adults with T2DM, circulating GLP‐1 levels predicted distinct patterns of hippocampal activation during memory encoding, suggesting that GLP‐1 signaling dynamically influences hippocampal circuit engagement in ways consistent with preclinical evidence of GLP‐1‐dependent synaptic plasticity (Canário et al. 2024). Additional mechanistic biomarker studies report improved insulin signaling and reduced plasma inflammatory markers following GLP‐1RA therapy, pointing toward systemic pathways that may underlie cognitive resilience (Gonzalez‐Rellan and Drucker 2025; Masson et al. 2024; Mashayekhi et al. 2024).

Early evidence from clinical trials suggested that GLP‐1RAs exert no significant effect on general cognitive performance, although potential benefits were observed in patients with T2DM younger than 65 years or those without cardiocerebrovascular disease history (Luan et al. 2022). More recent trials, however, report more encouraging findings. In individuals with mild Alzheimer's disease, liraglutide treatment has been associated with neuroprotection and reduced rates of cognitive decline by as much as 18% after 1 year of treatment compared to placebo. In a Phase 2b trial, liraglutide appears to slow atrophy in brain regions controlling memory, learning, language, and decision‐making by nearly 50% compared to placebo. Although these studies show improvements in some neurobiological markers (e.g., hippocampal connections, cerebral glucose metabolism, hippocampal activation on functional magnetic resonance imaging), such changes have not consistently correlated with improvements of cognitive performance measures. Nonetheless, converging epidemiological evidence suggests that GLP‐1RAs may confer protection against cognitive decline and potentially delaying progression to dementia in diabetic patients (Edison et al. 2025).

These promising findings help to prompt recent prospective Phase 3 trials directly testing semaglutide in humans with early AD. The global EVOKE (ClinicalTrials.gov identifiers: NCT04777396) and EVOKE+ (ClinicalTrials.gov identifiers: NCT04777409) studies were designed to assess whether semaglutide slows cognitive decline in people with mild cognitive impairment or mild dementia due to Alzheimer's pathology. Early biomarker analyses reportedly showed modest improvements in some Alzheimer's‐related biomarkers, but results announced in late 2025 did not demonstrate slowing of cognitive deterioration compared to placebo, leading to discontinuation of these specific programs (Novo Nordisk 2026). While these negative trial outcomes underscore the complexity of Alzheimer's pathophysiology and limitations of oral GLP‐1RAs as monotherapies for established disease, GLP‐1RAs may still hold promise in earlier disease stages, specific metabolic subgroups, injection‐based therapies to elevate bioavailability, or combination strategies targeting multiple pathological processes. Notably, much of the preclinical literature demonstrating central and behavioral effects has relied on liraglutide, which exhibits substantially greater brain penetrance than semaglutide (Abdulhameed et al. 2024). It may be that earlier GLP‐1RAs like liraglutide and dulaglutide (which showed the highest uptake in the hippocampus vs. 4 other radiolabeled GLP‐1RAs), while less effective for weight loss than newer GLP‐1RAs like semaglutide, may be better suited for AD based on greater direct access to hippocampal GLP‐1Rs.

Collectively, preclinical studies demonstrate robust neuroprotective and cognitive‐enhancing effects of GLP‐1 signaling through multiple mechanisms: neurotrophic support, synaptic plasticity enhancement, reduced neuroinflammation, and protection against AD pathology. Clinical studies have yielded more complex results, with population‐based studies and neuroimaging biomarker analyses supporting neuroprotective associations, while randomized controlled trials in established Alzheimer's disease have produced mixed outcomes. Ongoing research continues to investigate the therapeutic potential of GLP‐1RAs across different stages of cognitive impairment, metabolic subgroups, and combination treatment strategies. Future preclinical and clinical studies should evaluate the extent that GLP‐1RAs may differentially promote memory function in AD and other memory disorder models based on blood–brain barrier penetrance.

## 5 Discussion

The broad influence of GLP‐1 on cognitive processes is consistent with the widespread expression of its receptor across the CNS. Additionally, GLP‐1 is released upon nutrient sensing and serves as a potent indicator of caloric availability. Together, these features support a model in which GLP‐1R signaling modulates behavior and cognition, including motivation, affect, and memory, in accordance with the internal metabolic status. Such coupling is particularly beneficial for survival by enabling an organism to balance effort and rest based on actual energetic needs.

One clear consequence of this signaling is the suppression of reward‐driven behavior. GLP‐1R signaling dampens activity within the brain reward circuitry and consequently reduces effort‐based food‐seeking, a cost‐saving decision when energy balance is positive. The fact that these effects extend to addictive drugs in preclinical models suggests that, although the GLP‐1R system partially evolved to regulate feeding‐related motivation, its pharmacological activation can be leveraged to dampen other forms of motivated seeking that engage overlapping reward circuitry.

GLP‐1R signaling also shapes affective behavior in an energy‐dependent manner. Foraging for food involves exploration and risk‐taking, and by signaling nutrient availability, GLP‐1R signaling may reduce this intrinsic drive thereby biasing behavior away from exploration and toward conservation. Acutely, this shift can manifest as increased anxiety‐ and depressive‐like phenotypes in behavioral paradigms that rely on exploration and invigoration. Consistent with this interpretation, acute GLP‐1R activation engages stress‐related circuitry, reflecting a transient shift from external exploration toward internal regulation that may prepare the organism for the physiological demands associated with ingestion, including potential microbial or metabolic challenges that accompany food consumption. In contrast, long‐term treatment with GLP‐1RAs likely signals sustained energetic abundancy and thus is associated with neuroplastic adaptations that promote anxiolytic and antidepressant effects rather than a transient interoceptive response. This divergence between acute and chronic affective outcomes highlights treatment duration as a critical variable and underscores the importance of distinguishing state‐dependent behavioral effects from longer‐term adaptations when interpreting the emotional consequences of GLP‐1R signaling. It will be especially informative to observe the impact of GLP‐1RAs on affective symptoms while individuals are in the maintenance phase of treatment to determine how these symptoms evolve following treatment discontinuation.

GLP‐1R signaling also signifies that food has been successfully acquired and consumed. From a survival standpoint, it is advantageous to encode the environmental and contextual information surrounding successful feeding events to facilitate the initiation of future eating episodes. Consistent with this idea, GLP‐1R signaling enhances synaptic plasticity and learning and memory function in rodents, particularly within hippocampal circuits. Whether these cognitive benefits of GLP‐1RAs translate to humans likely depends on disease stage, treatment timing, and metabolic comorbidities.

Mechanistically, these behavioral and cognitive outcomes are likely mediated through multiple interacting pathways. Direct actions within the CNS are emerging as a key mechanism. In addition to accessing the brain via circumventricular organs lacking a functional BBB, such as the area postrema and the median eminence, GLP‐1R agonists can penetrate the BBB (Rhea et al. 2024) and may therefore directly engage GLP‐1Rs expressed throughout the central nervous system, including mesolimbic structures such as the VTA and ACB, hypothalamic nuclei such as the PVH, and hippocampal and cortical regions implicated in learning, memory, and emotional processing. Direct receptor activation within these circuits may modulate dopaminergic tone, synaptic plasticity, neuroinflammatory signaling, and neurotrophic pathways that influence reward valuation, motivation, and memory encoding. In parallel, GLP‐1R signaling on vagal afferent neurons provides a complementary peripheral neural pathway through which these agents may influence brain function. GLP‐1Rs are highly expressed in nodose ganglion neurons and on vagal afferent terminals innervating the gastrointestinal tract (Nakagawa et al. 2004; Vahl et al. 2007; Bucinskaite et al. 2009), and vagal signaling is well established to regulate metabolic homeostasis as well as the anorectic and incretin effects of endogenous GLP‐1 (Brierley and de Lartigue 2022; Krieger et al. 2015). Importantly, vagal circuits have been well implicated in reward processing and affective regulation (Décarie‐Spain et al. 2024), and gut‐derived vagal input also engages multi‐order brainstem–septal–hippocampal pathways that support memory formation (Décarie‐Spain et al. 2024; Suarez et al. 2018). Thus, activation of vagal GLP‐1Rs may influence cognition and behavior through ascending gut–brain pathways that converge on mesolimbic, hypothalamic, and hippocampal circuits. These indirect metabolic, direct central, and peripheral neural mechanisms are not mutually exclusive and likely operate in parallel in a context‐dependent manner. Finally, improvements in glucose metabolism and body weight reduction may indirectly enhance memory, mood, and reward‐related processes, as metabolic dysfunction, insulin resistance, and obesity are independently associated with impairments in executive function, affective regulation, and hippocampal‐dependent memory (Fulton et al. 2022; Convit et al. 2003; Farruggia and Small 2019). Normalization of metabolic state may therefore reduce obesity‐associated metabolic and inflammatory stress on neuronal circuits, complementing the direct effects of these agents.

Although GLP‐1R agonists exert effects on cognitive and neurobehavioral processes, this area of research remains comparatively nascent relative to the well‐established role of GLP‐1 in metabolic regulation and body weight control, and current evidence in cognition and psychiatry remains limited. Importantly, substantial interindividual variability characterizes metabolic responses to GLP‐1R‐based therapies, with a subset of patients exhibiting minimal body weight reduction (Squire et al. 2025). Whether similar non‐responder phenotypes exist with respect to putative cognitive, mood‐modulating, or anti‐addictive effects remains to be investigated.

Sex differences have been observed in glycemic control, weight reduction, and adverse event frequency, with women often demonstrating greater weight loss on GLP‐1RAs (Weiskirchen and Lonardo 2026; Yang et al. 2025; Börchers and Skibicka 2025; Rentzeperi et al. 2022). Yet inclusion of female subjects remains limited in many mechanistical preclinical studies, despite the growing popularity of this class of medication among women, highlighting fundamental gaps. Another central conceptual challenge is disentangling direct neuronal effects of GLP‐1R activation from the consequences of weight loss, improved glycemic control, reduced inflammation, or altered stress physiology. For instance, similar cognitive improvements have also been observed after bariatric procedures such as Roux‐en‐Y gastric bypass (Smith et al. 2020; Custers et al. 2024), underscoring the need for rigorously controlled designs including pair‐fed paradigms to isolate direct versus indirect mechanisms. Moreover, GLP‐1Rs are widely expressed across multiple brain regions, and only recently has research begun to define the region‐specific and circuit‐specific functions of these receptors beyond metabolic regulation. This anatomical breadth may explain why findings across reward, affective, and memory domains are variable in direction, magnitude, and mechanism, and at times, contradictory. While much of the mechanistic insight is derived from preclinical studies, clinical evidence remains sparse to date. Although a growing number of trials are beginning to evaluate cognitive endpoints, larger randomized clinical trials, together with carefully controlled translational studies sufficiently powered to evaluate cognitive processes, will be essential to clarify the neuropsychiatric potential of GLP‐1R agonists.

Collectively, the evidence reviewed here supports a model in which GLP‐1R signaling functions as a metabolic‐cognitive interface that aligns internal energetic state with behavioral output. Through coordinated effects on motivation, affect, and memory, GLP‐1R activation reduces unnecessary effort and risk, while facilitating progressive learning once energetic demands are met. The cognitive and emotional consequences of GLP‐1R signaling are highly context‐ and time‐dependent, shaped by treatment duration, metabolic state, and underlying interoceptive state and/or pathology. Future studies that explicitly consider these variables will be essential for fully defining the therapeutic potential and limitations of GLP‐1‐based interventions across metabolic, affective, and cognitive disorders.

## Author Contributions

S.X.G., L.D.‐S., C.G. and G.M. prepared the manuscript that was subsequently edited by S.E.K. and T.B. All authors approved the final version of the manuscript.

## Funding

This work was supported by the National Institutes of Health, DK104897, DK123423.

## Conflicts of Interest

T.B. receives research funding from Pfizer and Novo Nordisk that was not used in support of these studies. S.E.K. receives research funding from Novo Nordisk that was not used in support of these studies. All other authors declare no competing interests.

## Data Availability

The authors have nothing to report.
